# Altered Brain Functional Connectivity and Topological Structural in Girls with Idiopathic Central Precocious Puberty: A Graph Theory Analysis Based on Resting-State fMRI

**DOI:** 10.3390/children12050565

**Published:** 2025-04-27

**Authors:** Lu Tian, Yan Zeng, Helin Zheng, Jinhua Cai

**Affiliations:** 1 Department of Radiology, Children’s Hospital of Chongqing Medical University, National Clinical Research Center for Child Health and Disorders, Ministry of Education Key Laboratory of Child Development and Disorders, Chongqing Key Laboratory of Pediatric Metabolism and Inflammatory Diseases, Chongqing 400014, China; tianlukele@163.com (L.T.); 15923322936@163.com (H.Z.); 2 Department of Endocrinology, Children’s Hospital of Chongqing Medical University, National Clinical Research Center for Child Health and Disorders, Ministry of Education Key Laboratory of Child Development and Disorders, Chongqing Key Laboratory of Pediatric Metabolism and Inflammatory Diseases, Chongqing 400014, China; yanzeng1030@163.com

**Keywords:** idiopathic central precocious puberty, girl, brain functional connectivity, topological structural, complex networks graph theory analysis

## Abstract

Objectives: This study aimed to investigate changes in brain functional connectivity (FC) and topological structure in girls with idiopathic central precocious puberty (ICPP) using complex network theory analysis. Methods: Resting-state fMRI data from 53 ICPP girls (ages 6–8) and 51 controls were analysed. Graph theory was used to construct whole-brain functional networks, identify topological differences, and assess the relationship between sex hormone levels and network properties in regions with group differences. Results: RS-FC analysis revealed reduced connectivity in cognitive and emotional regulation regions in the ICPP group (*p* < 0.05), but enhanced connectivity in emotional perception and self-regulation areas, such as the amygdala and insula (*p* < 0.05), suggesting a compensatory mechanism. Graph theory showed that ICPP girls’ brain networks maintained small-world properties (γ > 1, λ ≈ 1, σ > 1). Local topological changes included decreased clustering and node efficiency in cognitive and emotional regulation regions, like the superior frontal gyrus and praecuneus (*p* < 0.05), while emotional regulation regions (amygdala, insula) showed increased clustering and node efficiency (*p* < 0.05), indicating compensation. Conclusions: This study highlights compensatory mechanisms in emotional regulation that may offset impairments in cognitive regions, offering new insights into ICPP’s neural mechanisms.

## 1. Introduction

Idiopathic central precocious puberty (ICPP) is defined as the premature activation of the hypothalamic–pituitary–gonadal (HPG) axis, occurring without a definitive aetiology or organic abnormality, and resulting in the development of secondary sexual characteristics in girls before the age of 8 and in boys before the age of 9 [1,2,3]. Precocious puberty occurs in girls 5 to 10 times more frequently than in boys, and over 90% of these cases are classified as idiopathic [4]. Approximately 10% of cases are classified as non-ICPP, which includes peripheral precocious puberty (PPP) and incomplete precocious puberty [4]. PPP, also known as pseudoprecocious puberty, presents with clinical features similar to ICPP, but unlike ICPP, it does not involve activation of the HPG axis. The source of sex hormones in PPP may be exogenous hormone intake or may originate from gonads or non-gonadal tumours and hyperplasia. Common causes include increased hormone secretion from the adrenal glands, ovaries, or testes, and associated conditions include congenital adrenal hyperplasia, autonomous ovarian cysts, oestrogen or androgen-secreting tumours, and McCune-Albright syndrome with precocious puberty, among others [1,2,3,4]. On the other hand, incomplete precocious puberty, also referred to as partial precocious puberty, typically manifests as isolated breast development, isolated early pubic hair appearance, or isolated early menarche. These conditions may resolve spontaneously after a period of time, although in a few cases, they may persist [1,2,3,4]. ICPP has significant impacts on children’s physical, psychological, and social development. Physiologically, ICPP is characterised by early breast development, accelerated bone maturation, and premature development of reproductive organs, which may lead to early epiphyseal closure and a reduced final adult height [5]. Furthermore, it is associated with an increased risk of conditions such as breast cancer, cervical cancer, and prostate cancer [6]. Psychologically, the early onset of puberty can cause significant emotional distress, leading to self-esteem issues, heightened anxiety, and identity confusion [7]. Moreover, these early psychological challenges may persist into adulthood. Socially, children with ICPP often face challenges in peer interactions, including feelings of exclusion, loneliness, and low self-worth. These difficulties are often exacerbated by bullying and social rejection, further contributing to psychological distress and negative emotional outcomes [8]. Despite significant progress in understanding its clinical manifestations, the pathogenesis of ICPP remains incompletely understood [9].

As the central organ of the endocrine system, the brain plays a key role in the pathogenesis of ICPP by regulating the secretion of sex hormones through the HPG axis. Consequently, the relationship between brain function and ICPP has become a key area of research. In routine clinical practice, patients with precocious puberty are typically evaluated using conventional cranial MRI to rule out structural abnormalities such as suprasellar arachnoid cysts and hypothalamic hamartomas [10]. In most cases of central precocious puberty (CPP), no significant abnormalities in the central nervous system are detected, and these cases are therefore classified as ICPP [10]. With the increasing application of functional MRI based on blood oxygen level-dependent (BOLD) contrast, resting-state fMRI has become a valuable method to assess intrinsic brain activity due to its advantages, including convenient data acquisition, a non-invasive approach to detecting changes in brain activity, and the availability of multiple brain activity parameters [11,12]. Recent studies have begun to explore brain functional changes in children with ICPP. Yu et al. [13] employed rs-fMRI with regional homogeneity (ReHo) analysis to explore the effects of early HPG axis activation on brain function in 41 ICPP patients and 44 controls. ReHo, as an important metric for assessing local synchronisation of brain activity, measures the similarity of BOLD signal time series among neighbouring voxels within a brain region, thereby indirectly reflecting the local synchronisation of neuronal activity [13]. ReHo provides a quantitative tool for identifying local brain functional abnormalities in ICPP. Compared to controls, the ICPP group exhibited increased ReHo in the left inferior temporal gyrus and decreased ReHo in the left superior temporal gyrus, bilateral orbitofrontal gyri, and left parieto-occipital gyrus. These findings suggest that early HPG axis activation may alter brain function in children with ICPP, potentially affecting cognitive and emotional processes. Chen et al. [14] investigated resting-state functional connectivity (RS-FC) differences between 29 ICPP patients and 38 controls, observing reduced RS-FC between the insulae and right middle frontal gyrus, as well as between the left fusiform gyrus and right amygdala. These results indicate that ICPP patients exhibit disrupted functional connectivity in specific brain regions, particularly those involved in cognitive and emotional regulation, which may affect their cognitive and emotional development. These findings indicate that early activation of the HPG axis impacts brain function, providing key insights into the brain function characteristics of ICPP girls. However, research on this topic is limited and still in its early stages, with most studies focusing on single analytical methods, such as ReHo or RS-FC [13,14], and lacking a systematic investigation of whole-brain network topology. Brain networks involve not only functional connectivity between regions but also the complex interactions between nodes and edges [15]. Therefore, it requires further in-depth analysis of multidimensional brain networks. Graph theory analysis is an effective approach for studying brain functional networks using imaging techniques, allowing for the examination of the topological properties of functional brain networks from a global perspective [15,16,17]. By conceptualizing the brain as a complex network composed of multiple functional regions (nodes) and their interconnections (edges), graph theory quantitatively evaluates the relationships between nodes, revealing both the global properties (such as small-worldness, normalised clustering coefficient, and normalised characteristic path length) and nodal properties (such as node efficiency and clustering coefficient) of the brain [16]. This method has been widely applied to the study of brain functional networks in both healthy individuals and patients [15,16,18,19,20]. However, the application of graph theory analysis to the study of ICPP brain networks remains an unexplored area in current research, warranting further investigation.

Additionally, it is important to consider the context of normal neurodevelopment, particularly as the developing brain in children follows specific trajectories characterised by progressive changes in functional connectivity, synaptic pruning, and network specialisation. These processes may interact with the effects of early hormonal activation.

This study is the first to systematically apply complex network graph theory analysis to investigate alterations in brain functional connectivity and topological organisation in girls with ICPP.

By assessing both global and nodal topological properties of the whole-brain functional network and exploring the relationship between sex hormone levels and network topological properties in brain regions with significant group differences, this study provides novel insights into the neural mechanisms underlying early HPG axis activation. Importantly, by revealing specific alterations in brain network organisation associated with hormonal changes, our findings contribute to a more comprehensive understanding of the neurobiological basis of ICPP. These results may facilitate the identification of potential neuroimaging biomarkers for early diagnosis and offer guidance for timely and targeted clinical interventions.

## 2. Methods

**Figure 1:**
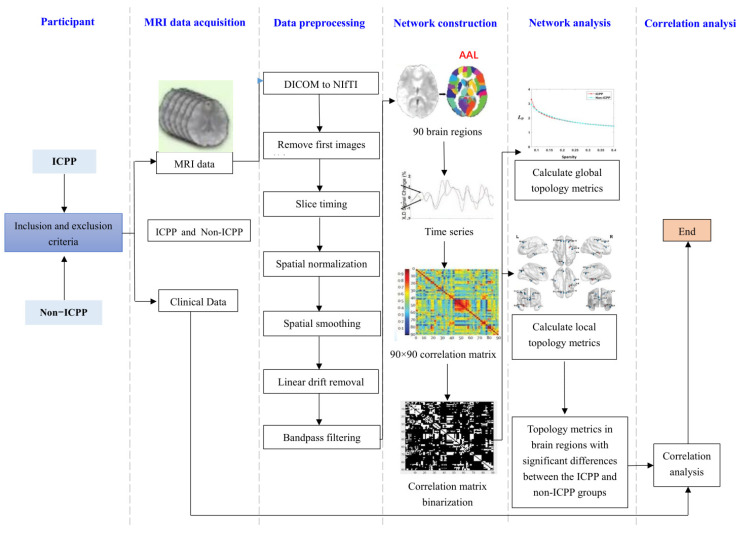
Flow chart of graph theory analysis.

The overall flowchart of the research method is shown in Figure 1.

### 2.1. Participants

This study included 104 female children diagnosed with precocious puberty who presented to the endocrinology department of our hospital between January 2023 and January 2025 with clinical manifestations of secondary sexual characteristics, such as breast development or vaginal bleeding. A comprehensive evaluation was conducted based on established guidelines and clinical pathways for diagnosing precocious puberty. Using the gold-standard gonadotropin-releasing hormone (GnRH) stimulation test, patients were classified into two groups: ICPP and non-ICPP. The ICPP group consisted of 53 patients aged 6–8 years (mean age: 6.86 ± 1.01 years), while the control group, matched for age and sample size, included 51 non-ICPP patients aged 6–8 years (mean age: 6.54 ± 1.31 years).

The inclusion criteria for the ICPP group were as follows: (1) bone age exceeding chronological age by at least one year; (2) breast development at Tanner stage ≥2; (3) body mass index (BMI) within the 25th to 85th percentile for age and sex; (4) normal brain and pituitary MRI findings; and (5) GnRH stimulation test results confirming HPG axis activation. The GnRH stimulation test was performed between 8:00 and 9:00 AM. Patients received an intravenous injection of a GnRH analogue (GnRHa, gonadorelin) at a dose of 2.5 μg/kg (maximum dose: 100 μg). Serum luteinizing hormone (LH) and follicle-stimulating hormone (FSH) levels were measured at 0, 30, 60, and 90 min post-injection using chemiluminescence immunoassay. Activation of the HPG axis, confirming ICPP, was defined as a peak LH level ≥5.0 U/L and a peak LH/FSH ratio ≥0.6 [21,22]. For those who did not meet this criterion, they were diagnosed with GnRH-independent precocious puberty, referred to as non-ICPP within this study. The inclusion criteria for the non-ICPP group mirrored those for ICPP, except that the GnRH stimulation test results indicated no activation of the HPG axis. Exclusion criteria for both groups were as follows: (1) left-handedness; (2) premature birth; (3) advanced pubertal status; (4) precocious puberty due to central nervous system injury or congenital factors; (5) Previously diagnosed psychiatric or neurodevelopmental disorders, such as attention deficit hyperactivity disorder (ADHD), autism spectrum disorder (ASD), major depressive disorder, or anxiety disorders requiring clinical intervention or medication; (6) prior sex hormone therapy; (7) MRI contraindications; and (8) excessive head motion or artifacts that interfered with imaging quality.

### 2.2. Clinical Data

Clinical data collection was meticulously guided by a comprehensive literature review and consultations with clinical experts in the field of endocrinology. Data acquisition was categorised into four primary domains: general parameters, clinical parameters, laboratory parameters, and imaging parameters. General and clinical parameters included chronological age, height, weight, BMI, and clinical manifestations. Biochemical indicators comprised basal and peak serum levels of follicle-stimulating hormone (FSH) and luteinizing hormone (LH), along with oestradiol levels. Imaging parameters were derived from pelvic ultrasonography, which measured the largest longitudinal, anteroposterior, and transverse diameters of the uterus and bilateral ovaries. Uterine and ovarian volumes were calculated using the ellipsoid formula [23]: *V* = *D1* × *D2* × *D3* × 0.523, where *D1* is the largest longitudinal diameter, *D2* the largest anteroposterior diameter, and *D3* the largest transverse diameter. Bone age (BA) was assessed using left-hand X-ray imaging and analysed according to the Tanner-Whitehouse 2 (TW2) method [24]. All participants underwent basic psychological assessments using the Child Behaviour Checklist (CBCL), the Chinese Revision of the Wechsler Intelligence Scale for Children (WISC-CR), and the Hamilton Anxiety Scale (HAMA) to evaluate emotional and cognitive status.

### 2.3. MRI Data Acquisition

All MRI data were acquired using a Philips Achieva 3.0T (Philips Healthcare, Amsterdam, Netherlands)superconducting magnetic resonance scanner equipped with an 8-channel orthogonal phased array head coil for signal reception. Participants were instructed to prepare for the scan by removing all ferromagnetic items such as jewellery, mobile phones, coins, watches, and keys. During the scan, participants lay supine with their heads immobilised using foam pads and straps provided by Philips to minimise motion. Earplugs were used to reduce auditory disturbances caused by scanner noise. Each participant underwent a routine MR scan with sequences including axial T1WI, T2WI, T2FLAIR, and sagittal T2WI to exclude obvious organic neurological disease. Following the routine MR scan, an rs-fMRI sequence was acquired using an echo-planar imaging (EPI) sequence. The parameters were as follows: TR = 2000 ms, TE = 35 ms, slice thickness = 4 mm, slices = 33, FOV = 240 mm × 240 mm, acquisition matrix = 80 × 78, flip angle = 90°, voxel size = 3.75 mm × 3.75 mm × 4 mm with no gap, and total acquisition time = 8 min 06 s.

T1-weighted structural images were acquired using a turbo field echo (TFE) gradient-echo sequence with the following parameters: repetition time (TR) = 7.4 ms, echo time (TE) = 3.8 ms, slice thickness = 1 mm, interslice gap = 0 mm, total of 260 slices; field of view (FOV) = 250 mm × 250 mm × 156 mm; acquisition matrix = 228 × 227; number of excitations (NEX) = 1; flip angle = 8°; voxel size = 1.1 mm × 1.1 mm × 1.1 mm; total acquisition time = 4 min 16 s.

### 2.4. Data Preprocessing

The fMRI data were pre-processed using Statistical Parametric Mapping (SPM12, http://www.fil.ion.ucl.ac.uk/spm/software/spm12, accessed on 15 October 2024) in conjunction with the GRETNA toolbox (version 2.0, https://github.com/sandywang/GRETNA, accessed on 15 October 2024), following the workflow outlined below: (1) DICOM to NIFTI conversion: dicom images were converted to NIFTI format. To stabilise the MRI signal, the first 10 time points were discarded. (2) Slice timing and motion correction: the remaining images underwent slice timing correction to account for temporal discrepancies across slices and were then subjected to head motion correction. Scans exhibiting translation > 2 mm or rotation > 2 degrees after correction were excluded from further analysis. (3) Spatial normalisation: co-registration of T1-weighted structural images and segmentation was performed for spatial normalisation. Resting-state functional data were resampled to a uniform voxel size of 3 mm × 3 mm × 3 mm. (4) Spatial smoothing: a Gaussian kernel with a full-width at half-maximum (FWHM) of 6 mm was applied to smooth the standardised imaging data, enhancing signal-to-noise ratio and comparability across subjects. (5) Linear drift removal: Linear regression was employed to remove signal drifts caused by thermal noise, including that generated by the MRI scanner. (6) Bandpass filtering: temporal signals were filtered within a frequency range of 0.01–0.08 Hz to isolate relevant neural signals while mitigating physiological noise.

### 2.5. Network Construction

The functional brain network was constructed and topological properties were analysed using GRETNA v2.0.0 software (http://www.nitrc.org/projects/gretna/, accessed on 15 October 2024) with the automated anatomical labelling (AAL) template. This atlas divides the brain into 90 regions, each treated as a network node. The time series for each ROI was averaged to define the edges, resulting in a 90 × 90 correlation matrix. To facilitate parametric statistical analysis, Fisher’s r-to-z transformation was applied to the correlation matrices to enhance normality. The correlation matrices were binarised using a sparsity thresholding method to optimise network properties. The sparsity range was set from 0.08 to 0.40, with increments of 0.01, balancing the estimation of small-world characteristics while minimising false connections in the network. Within this sparsity range, the area under the curve (AUC) of global and nodal topological attributes was calculated. This approach provided a comprehensive statistical description of network properties while reducing potential bias introduced by any single threshold value. It enabled a robust evaluation of functional connectivity and network topology while ensuring methodological rigor.

### 2.6. Network Analysis

After constructing the functional brain network, global and nodal topological metrics were calculated for both the ICPP and non-ICPP groups. Global properties assessed included clustering coefficient (*C*_p_), characteristic path length (*L*_p_), normalised clustering coefficient (γ), normalised characteristic path length (λ), small-worldness (σ), global efficiency (*E*_glob_), and local efficiency (*E*_loc_). Nodal properties analysed comprised degree centrality (*D*_c_), nodal efficiency (*N*_e_), and nodal clustering coefficient (*N*_cp_), which were also computed. A network that meets the criteria γ > 1, λ ≈ 1, and σ > 1 is recognised as a small-world network.

### 2.7. Statistical Analysis

Statistical analyses of demographic and clinical data between the two groups were conducted using SPSS version 24.0 (IBM Inc., Armonk, NY, USA). Data normality was assessed using the Shapiro–Wilk test. Normally distributed continuous variables were presented as mean ± standard deviation ($\bar{x}$ ± S), and group comparisons were performed using an independent sample *t*-test. Non-normally distributed variables were expressed as medians with interquartile ranges [M (P25, P75)], with group comparisons conducted using the Mann–Whitney U test. Categorical variables were reported as rates or proportions, and group differences were evaluated using the chi-square test or Fisher’s exact test, as appropriate.

Differences in functional connectivity between brain regions were analysed using the connection module of the GRETNA software. Group differences in functional connections were identified using a two-sample *t*-test, with false discovery rate (FDR) correction applied to control for multiple comparisons. Significant results were visualised using Brainnet Viewer (http://www.nitrc.org/projects/bnv, accessed on 27 April 2024).

The topological properties of brain networks were analysed with GRETNA software. The AUC of topological metrics was statistically analysed, and group differences in global and nodal properties were assessed using two-sample *t*-tests with FDR correction. Bonferroni correction was applied to control for multiple comparisons of nodal properties. Significant nodal differences were displayed using Brainnet Viewer.

Finally, partial correlation analysis examined the associations between significantly different topological properties and sex hormone levels, adjusting for age and gender as covariates. A *p*-value < 0.05 was considered statistically significant.

## 3. Results

### 3.1. Demographics and Characteristics

The demographic and clinical characteristics of the study participants are summarised in Table 1. The Shapiro–Wilk test indicated that actual age, height, and weight in both groups followed a normal distribution and were therefore expressed as mean ± standard deviation ($\bar{x}$ ± S). Conversely, all other parameters did not follow a normal distribution (*p* < 0.05) and were presented as median and interquartile range [M (P25, P75)]. As detailed in Table 1, ten parameters differed significantly between the ICPP and non-ICPP groups (*p* < 0.05), including height, weight, baseline LH, baseline FSH, LH peak, LH peak/FSH peak ratio, bone age, left ovarian volume, right ovarian volume, and uterine volume. Specifically, general parameters showed that the ICPP group had significantly greater height and weight than the non-ICPP group (*p* < 0.05). Biochemical indicators showed that baseline FSH, baseline LH, LH peak, and the LH peak/FSH peak ratio were significantly higher in the ICPP group than in the non-ICPP group. Imaging parameters showed that bone age, bilateral ovarian volumes, and uterine volume were significantly higher in the ICPP group (*p* < 0.05), highlighting notable structural and developmental differences between the two cohorts. The scale results indicate that premature activation of the HPG axis does not have significant or substantial adverse effects on the cognitive and emotional development of girls aged 6 to 8 years. However, our findings do not rule out the possibility of significant behavioural abnormalities emerging after prolonged exposure to a prematurely activated HPG axis. Close attention should be paid to the mental health of ICPP patients.

### 3.2. Comparison of RS-FC Between Two Groups

**Figure 2:**
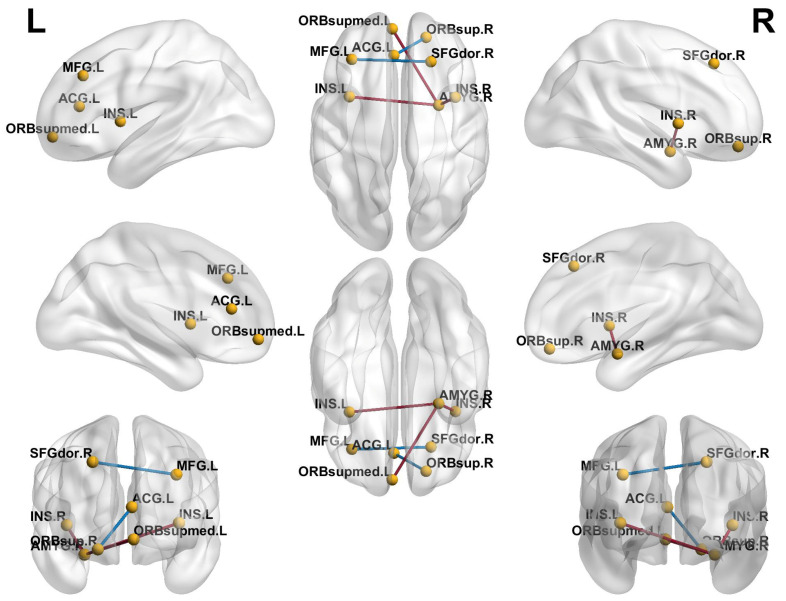
Brain regions showing differences in resting-state functional connectivity between the ICPP and non-ICPP groups. Note: The yellow dots represent brain regions, the red lines represent enhanced functional connections between the two brain regions, and the blue lines represent reduced functional connections between the two brain regions.rain regions. SFGdor.R, right dorsolateral superior frontal gyrus; ORBsup.R, right orbital superior frontal gyrus; MFG.L, left medial frontal gyrus; ORBsupmed.L, left medial orbital superior frontal gyrus; INS.L, left insula, INS.R, right insula; ACG.L, left anterior cingulate gyrus; AMYG.R, right amygdala.

The connection analysis performed using GRETNA software identified significant differences in resting-state functional connectivity between the ICPP and non-ICPP groups, encompassing five functional connections and eight brain region nodes (t = 3.524, *p* < 0.001). The implicated nodes included the right dorsolateral superior frontal gyrus, right orbital superior frontal gyrus, left medial frontal gyrus, left medial orbital superior frontal gyrus, left insula, right insula, left anterior cingulate gyrus, and right amygdala (Figure 2). Compared to the non-ICPP group, the ICPP group exhibited significantly reduced functional connectivity between the right dorsolateral superior frontal gyrus and the left middle frontal gyrus, as well as between the right orbital superior frontal gyrus and the left anterior cingulate gyrus (t = −3.623 and −4.163, *p* < 0.05). Conversely, the ICPP group showed increased functional connectivity between the right amygdala and the bilateral insulae, as well as between the right amygdala and the left orbital superior frontal gyrus (t = 3.576, 4.359, and 3.534; all *p* < 0.05).

### 3.3. Comparison of Global Topological Properties Between Two Groups

**Figure 3:**
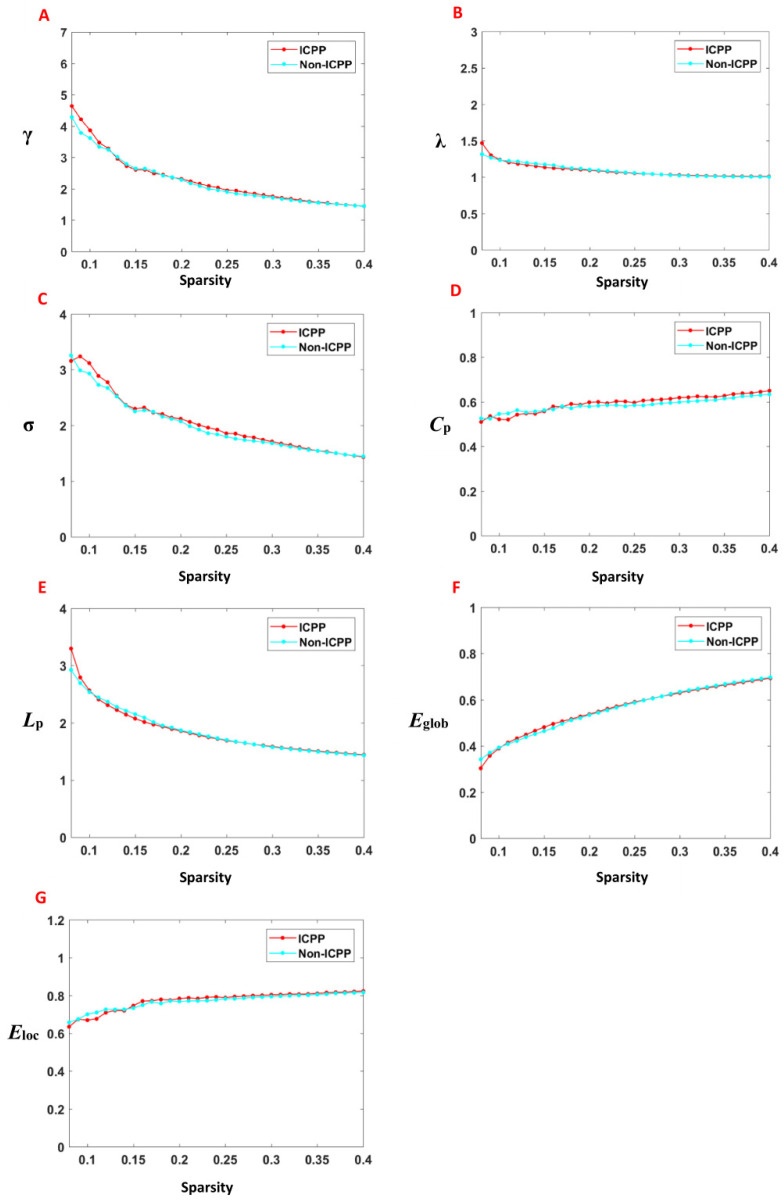
The variation of the global properties of the brain functional network with the threshold value and the area under the curve. Note: γ, normalised clustering coefficient; λ, normalised characteristic path length; σ, small worldness; *C*_p_, clustering coefficient; *L*_p_, characteristic path length; *E*_glob_, global efficiency; *E*_loc_, local efficiency; (**A**–**G**) is the γ, λ, σ, *C*_p_, *L*_p_, *E*_glob_, and *E*_loc_ with the change of thresholds. The solid line represents the mean value under different thresholds. The networks of both groups exhibit typical small-world topological properties, i.e., γ > 1, λ ≈ 1, and σ > 1.

Within the predefined network sparsity thresholds (0.08–0.40), the functional brain networks in both groups demonstrated typical small-world properties, characterised by γ > 1, λ ≈ 1, and σ > 1. Across this range, no statistically significant differences were observed in the AUC for key network metrics, including clustering coefficient (*C*_p_), characteristic path length (*L*_p_), normalised clustering coefficient (γ), normalised characteristic path length (λ), small-world index (σ), global efficiency (*E*_glob_), and local efficiency (*E*_loc_), between the ICPP and non-ICPP groups (*p* > 0.05) (Figure 3, Table 2).

### 3.4. Comparison of Local Topological Properties Between Two Groups

**Figure 4:**
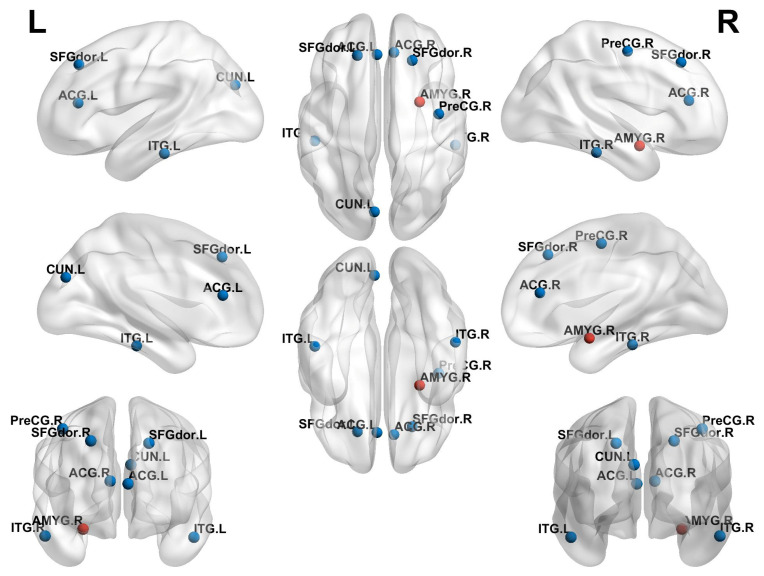
Brain regions with statistically significant differences in nodal clustering coefficients between the ICPP and non-ICPP groups. Note: Red dots represent brain regions with increased clustering coefficients, and blue dots represent brain regions with decreased clustering coefficients. SFGdor.L, left dorsolateral superior frontal gyrus; SFGdor.R, right dorsolateral superior frontal gyrus; ACG.L, left anterior cingulate gyrus; ACG.R, right anterior cingulate gyrus; AMYG.R, right amygdala; CUN.L, left praecuneus; ITG.L, left inferior temporal gyrus; ITG.R, right inferior temporal gyrus.

**Figure 5:**
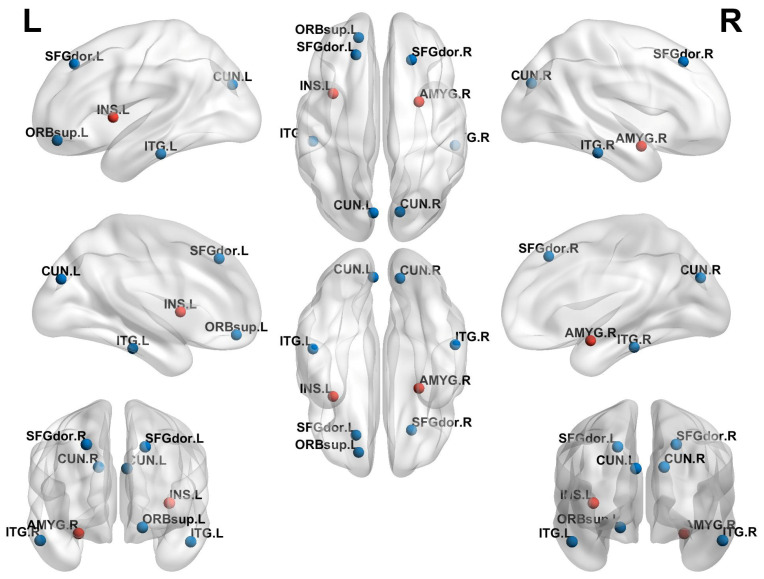
Brain regions with statistically significant differences in nodal efficiency between the ICPP and non-ICPP groups. Note: Red dots represent brain regions with increased nodal efficiency, and blue dots represent brain regions with decreased nodal efficiency. SFGdor.L, left dorsolateral superior frontal gyrus; SFGdor.R, right dorsolateral superior frontal gyrus; ORBsup.L, left orbital superior frontal gyrus; INS.L, left insula; AMYG.R, right amygdala; CUN.L, left praecuneus; CUN.R, right praecuneus; ITG.L, left inferior temporal gyrus; ITG.R, right inferior temporal gyrus.

Compared to the non-ICPP group, the ICPP group exhibited significant alterations in node clustering coefficient and node efficiency (*p* < 0.05, FDR correction). Specifically, the ICPP group demonstrated decreased clustering coefficients in the left and right dorsolateral superior frontal gyrus, left and right anterior cingulate gyrus, left precuneus, and left and right inferior temporal gyrus. In contrast, the clustering coefficient of the right amygdala was significantly increased (Figure 4, Table 3). Similarly, node efficiency was reduced in the left and right dorsolateral superior frontal gyrus, left orbital superior frontal gyrus, left and right precuneus, and left and right inferior temporal gyrus in the ICPP group compared to the non-ICPP group. In contrast, increased node efficiency was observed in the left insula and right amygdala (Figure 5, Table 4).

### 3.5. Relationships Between Sex Hormone and Topological Properties

**Figure 6:**
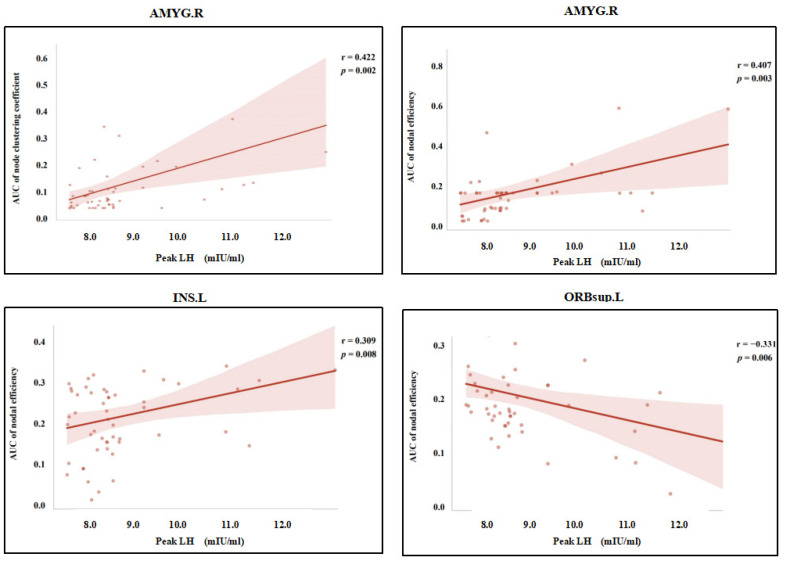
Scatter plot of the correlation analysis between the AUC values of local topological properties in brain regions with significant differences between the ICPP and non-ICPP groups and sex hormones. *Note:* AMYG.R, right amygdala; INS.L, left insula; ORBsup.L, left orbital superior frontal gyrus; AUC, area under the curve; The statistical significance level was set as *p* < 0.05.

The correlation analysis revealed that the clustering coefficient and node efficiency of the right amygdala were positively correlated with peak LH levels (r = 0.422, *p* = 0.002; r = 0.407, *p* = 0.003). Similarly, the node efficiency of the left insula also showed a positive correlation with LH peak levels (r = 0.309, *p* = 0.008). In contrast, the node efficiency of the left orbital superior frontal gyrus was negatively correlated with LH peak levels (r = −0.331, *p* = 0.006), as shown in Figure 6. Correlations between other topological properties and sex hormone levels were not statistically significant (*p* > 0.05).

## 4. Discussion

To our knowledge, this study is the first to explore brain functional connectivity and topological changes in girls with ICPP through complex network graph theory analysis, filling the gap in previous research that relied on single analytical methods (such as ReHo or RS-FC) [13,14], which were unable to comprehensively reveal whole-brain network topological features.

Through RS-FC analysis, we not only identified alterations in FC in brain regions associated with cognition and emotional regulation in ICPP patients but also uncovered potential compensatory mechanisms in brain function. Compared with the non-ICPP group, the ICPP group exhibited reduced FC between the right dorsolateral superior frontal gyrus and the left middle frontal gyrus, as well as between the right orbital superior frontal gyrus and the left anterior cingulate gyrus. Conversely, the ICPP group demonstrated significantly enhanced FC between the right amygdala and the bilateral insulae, as well as between the right amygdala and the left orbital superior frontal gyrus. The dorsolateral superior frontal gyrus and middle frontal gyrus are primarily involved in executive functions, working memory, and advanced cognitive regulation [25,26], whereas the orbital superior frontal gyrus and anterior cingulate gyrus play crucial roles in emotional regulation and decision-making [27]. The observed reductions in functional connectivity between these regions may indicate potential impairments in advanced cognitive functions and emotional regulation capabilities in ICPP patients. This finding is consistent with previous studies [14,28], which have also reported diminished functional connectivity in key brain regions associated with cognition and emotion regulation in girls with ICPP. For instance, the study by Chen et al. [14] reported reduced functional connectivity between the left and right insula and the right middle frontal gyrus in the ICPP group, suggesting that weakened connectivity between the insula and advanced cognitive regions may negatively impact cognitive performance in these patients. Similarly, the decrease in functional connectivity between the left fusiform gyrus and the right amygdala may reflect disturbances in emotional regulation. Qin et al. [28] observed reduced functional connectivity between the parietal and occipital cortices (e.g., bilateral superior parietal lobule and left superior occipital gyrus) and visual-related regions (right calcarine sulcus) in girls with CPP using resting-state fMRI, further supporting the hypothesis that premature activation of the HPG axis may mediate the risk of cognitive impairment. In contrast to previous studies that primarily report reduced functional connectivity in cognitive and emotional networks in ICPP patients, our analysis is the first to identify enhanced functional connectivity in emotion-related brain regions (such as the amygdala [29] and insula [30]) in these patients. This phenomenon may reflect a compensatory mechanism that emerges as a part of the ongoing neurodevelopmental process in ICPP patients. Given the heightened neural plasticity in adolescence, the brain may engage in adaptive responses to maintain cognitive and emotional regulation when key networks show reduced efficiency. Specifically, when connectivity in higher-order cognitive networks decreases, which could impact executive functions and memory processing, ICPP patients may compensate by enhancing functional connectivity in emotion-related brain regions, such as the amygdala and insula. This compensatory strategy might help preserve the ability to regulate emotions, process social cognition, and manage reward processing despite the cognitive deficits. From a neurodevelopmental perspective, this compensatory mechanism may reflect a transient response to disruption of the normal developmental trajectory caused by early activation of the HCG axis, and its long-term developmental consequences deserve further exploration, and it warrants further exploration to understand its long-term developmental consequences. The strengthened amygdala-insula-orbital frontal gyrus network connectivity may indicate that the brain compensates for cognitive and emotional function deficits by improving the efficiency of emotional regulation under increased cognitive load. Through RS-FC analysis, this study offers a novel perspective on the functional connectivity changes in cognitive function and emotional regulation in ICPP girls, and for the first time, reveals potential compensatory mechanisms in brain function in ICPP patients.

Moreover, graph theory analysis indicates that the brain networks of ICPP girls retain a “small-world network” characteristic [31], reflecting the robust organisation of brain connectivity that supports efficient information transfer while minimising energy consumption—a hallmark of effective neurodevelopmental adaptation. However, certain brain regions, such as the bilateral dorsolateral superior frontal gyrus, anterior cingulate gyrus, precuneus, and inferior temporal gyrus, exhibit decreased clustering coefficients and node efficiency. These areas are central to higher-order cognition, task execution, and emotional regulation. This suggests that while the brain retains overall efficiency, local disruptions in network integration may hinder the developmental progress of complex cognitive and emotional functions. These findings highlight the nuanced balance between adaptive and maladaptive changes in brain networks during the developmental stages of ICPP. For example, the dorsolateral superior frontal gyrus is central to executive functions and cognitive control, and a decline in its integration capacity may impair the execution of complex tasks, such as planning, decision-making, and conflict monitoring [32]. The anterior cingulate gyrus is involved in emotional regulation and cognitive monitoring, and a decline in its function may weaken emotional regulation capabilities [33]. The precuneus is closely related to information integration, self-referential processing, and visuospatial cognition, and its diminished function may affect the integration of complex cognitive tasks [34]. The inferior temporal gyrus, which is involved in semantic memory and language processing [35], also shows decreased local and global connectivity efficiency, suggesting that language comprehension and semantic integration may be affected. However, areas related to emotional perception and self-regulation, such as the amygdala and insula [30], show enhanced clustering coefficients and node efficiency, indicating that ICPP patients may compensate for dysfunction in other regions through compensatory mechanisms. The enhancement of the amygdala in emotional processing reflects an improved ability in emotional regulation and stress response, while the enhancement of the insula suggests an increase in emotional perception, self-awareness, and physiological regulation to cope with deficits in other brain areas. In the short term, this compensatory mechanism may serve to stabilise emotional regulation and cognitive function despite disruptions in higher-order networks. However, given the critical period of brain development during adolescence, prolonged reliance on these compensatory mechanisms may lead to maladaptive patterns of brain resource allocation. Over time, this imbalance could compromise the full maturation of cognitive, emotional, and social processing, potentially hindering optimal neurodevelopment. Therefore, future studies should investigate the long-term consequences of these compensatory responses, with a particular focus on their effects on the trajectory of cognitive, emotional, and social development in ICPP patients as they transition into adulthood.

Additionally, the underlying cause of the aforementioned changes in girls with ICPP warrants further investigation. Based on existing literature and our findings, we propose the following hypothesis: these changes in ICPP girls may be associated with the early activation of the HPG axis, which leads to fluctuations in sex hormone levels, such as LH and FSH. Previous studies have suggested that early activation of the HPG axis may result in abnormal functional connectivity in brain regions associated with emotion and cognition, thereby affecting brain function in ICPP girls [14,28]. Our correlation analysis results further support this hypothesis, showing that the clustering coefficient and node efficiency of the right amygdala, as well as the node efficiency of the left insula, are positively correlated with peak LH levels. In contrast, the node efficiency of the left dorsolateral prefrontal cortex is negatively correlated with peak LH levels. These findings provide additional evidence for the direct regulatory effect of early activation of the HPG axis, leading to elevated LH peaks, on brain network topology, further supporting our proposed hypothesis.

In summary, these findings contribute to a deeper understanding of how early activation of the HPG axis affects brain network development and provide theoretical support for compensatory mechanisms in emotional regulation.

## 5. Limitations

This study has several limitations. Firstly, the cross-sectional design of this study limits our ability to definitively establish a causal relationship between brain connectivity changes and the onset of ICPP. While we hypothesise that these changes may be closely related to ICPP, we cannot determine whether they are causal or consequential. Future longitudinal studies will be essential to investigate whether these brain connectivity changes are permanent or whether treatment with LHRH analogues leads to a regression of these findings. If these changes reverse after treatment, it will support the hypothesis that they are a consequence of ICPP; conversely, if the changes persist, it may suggest a causal role in the development of ICPP. Therefore, we recommend conducting longitudinal follow-up studies to better understand the role of brain connectivity in ICPP and clarify the potential causal relationship. Secondly, due to the significantly higher prevalence of ICPP in girls compared to boys, and to avoid potential confounding effects of gender differences, this study only included girls as participants and did not consider a male group. This limitation somewhat restricts our comprehensive understanding of gender differences in ICPP. Future research should include male samples to more fully explore the impact of gender on brain function in ICPP and its clinical implications.

## 6. Conclusions

This study is the first to explore brain functional connectivity and topological changes in girls with ICPP using complex network graph theory analysis. RS-FC analysis shows that, while functional connectivity in brain regions associated with cognitive regulation decreases, there is a compensatory enhancement in brain regions related to emotional regulation. Graph theory analysis further reveals that, although the overall network functional efficiency is maintained, key brain regions associated with higher cognitive functions and emotional regulation are impaired. The enhanced function in emotional regulation regions may compensate for the deficits in other brain areas. Additionally, correlation analysis indicates a close relationship between peak LH levels and the network function of brain regions related to emotional regulation and cognitive functions. These findings contribute to a deeper understanding of how early activation of the HPG axis affects brain network development and provide theoretical support for compensatory mechanisms in emotional regulation.

## Figures and Tables

**Table 1:** Demographic and clinical characteristics.

Parameters	ICPP Group (n = 53)	Non-ICPP Group (n = 51)	Statistic	*p*
General parameters				
CA (year)	6.86 ± 1.01	6.54 ± 1.31	1.549 ^a^	0.121
Height (cm)	128.78 ± 6.90	119.11 ± 6.11	3.558 ^a^	<0.001
Weight (kg)	29.01 ± 6.08	24.81 ± 4.32	3.924 ^a^	<0.001
BMI (kg/m^2^)	17.79 (16.14, 19.07)	16.76 (15.06, 18.5)	1.468 ^c^	0.143
Clinical manifestation				
Breast development	45 (85%)	42 (82%)	1.814 ^b^	0.070
Vaginal bleeding	21 (39%)	18 (35%)	1.549 ^b^	0.080
Biochemical indicators				
Basal LH (mIU/mL)	0.24 (0.14, 0.43)	0.10 (0.10, 0.12)	2.033 ^c^	<0.001
Basal FSH (mIU/m)	2.61 (1.69, 3.52)	1.85 (1.24, 2.44)	3.110 ^c^	0.002
Peak LH (mIU/mL)	10.80 (8.22, 12.69)	3.38 (2.17, 4.84)	8.043 ^c^	<0.001
Peak FSH (mIU/mL)	11.48 (9.56, 12.60)	11.38 (9.43, 13.28)	1.278 ^c^	0.095
Peak LH/Peak FSH	0.94 (0.85, 1.05)	0.29 (0.23, 0.36)	9.241 ^c^	<0.001
E2 (pmol/L)	78.50 (73.40, 82.00)	73.40 (73.40, 82.01)	2.238 ^c^	0.055
Imaging parameters				
BA (years)	9.2 (8.40, 9.60)	8.30 (7.40, 9.30)	3.186 ^c^	0.001
Left ovarian volume (cm^3^)	1.34 (1.01, 1.96)	0.86 (0.50, 1.61)	3.161 ^c^	<0.001
Right ovarian volume (cm^3^)	1.31 (0.98, 1.87)	0.88 (0.58, 1.66)	3.124 ^a^	<0.001
Uterine volume (cm^3^)	2.63 (2.26, 3.12)	2.24 (1.63, 2.86)	2.245 ^c^	0.003
Scale				
WISC-CR (IQ) score	104.12 ± 10.28	102.25 ± 13.96	1.428 ^a^	0.820
CBCL score	7.90 ± 6.40	8.10 ± 5.87	1.338 ^a^	0.736
HAMA score	5.23 ± 1.21	5.42 ± 1.00	1.268 ^a^	0.870

Note: n, number of patients; ICPP, idiopathic central precocious puberty; CA, chronologic age; BMI, body mass index; LH, luteinizing hormone; FSH, follicle-stimulation hormone; E2, oestradiol; BA, bone age; WISC-CR, Wechsler intelligence scale for children-revised chinese revision; CBCL, child behaviour checklist; HAMA, Hamilton anxiety rating scale; ^a^, two-sample *t*-test for normally distributed data; ^b^, chi-square test for classifying categorical variables; ^c^, Mann–Whitney u-test for nonnormally distributed data; The results were considered statistically significant at *p* < 0.05.

**Table 2:** Comparison of the global topological properties of brain functional networks between the ICPP and non-ICPP groups.

Group	n	γ	λ	σ	*C*_p_	*L*_p_	*E*_glob_	*E*_loc_
ICPP	53	0.60 ± 0.12	0.35 ± 0.02	0.54 ± 0.11	0.19 ± 0.01	0.60 ± 0.04	0.178 ± 0.08	0.24 ± 0.01
Non-ICPP	51	0.60 ± 0.12	0.36 ± 0.02	0.53 ± 0.12	0.20 ± 0.02	0.62 ± 0.05	0.17 ± 0.01	0.25 ± 0.01
T		0.10	−1.14	0.34	−1.78	−1.20	1.43	−1.65
*p*		0.92	0.27	0.74	0.09	0.24	0.16	0.12

Note: n, number of patients; ICPP, idiopathic central precocious puberty; γ, normalised clustering coefficient; λ, normalised characteristic path length; σ, small worldness; *C*_p_, clustering coefficient; *L*_p_, characteristic path length; *E*_glob_, global efficiency; *E*_loc_, local efficiency; The statistically significant level was set as *p* < 0.05.

**Table 3:** Brain regions with statistically significant differences in nodal clustering coefficients between the ICPP and non-ICPP groups.

AAL No.	Region	MNI Coordinates			T	*p*
		X	Y	Z		
3	SFGdor.L	−22	18	72	−3.4474	0.0010
4	SFGdor.R	24	20	70	−2.1161	0.0437
31	ACG.L	−6	40	2	−2.1415	0.0414
32	ACG.R	8	42	4	−2.0243	0.0410
42	AMYG.R	24	−2	−14	2.2437	0.0333
45	CUN.L	−8	−76	20	−2.2693	0.0315
89	ITG.L	−50	−40	−20	−2.2247	0.0347
90	ITG.R	52	−38	−20	−2.7442	0.0107

Note: AAL No., automated anatomical labelling number; MNI, Montreal Neurological Institute; SFGdor.L, left dorsolateral superior frontal gyrus; SFGdor.R, right dorsolateral superior frontal gyrus; ACG.L, left anterior cingulate gyrus; ACG.R, right anterior cingulate gyrus; AMYG.R, right amygdala; CUN.L, left precuneus; ITG.L, left inferior temporal gyrus; ITG.R, right inferior temporal gyrus; The statistically significant level was set as *p* < 0.05.

**Table 4:** Brain regions with statistically significant differences in nodal efficiency between the ICPP and non-ICPP groups.

AAL No.	Region	MNI Coordinates			T	*p*
		X	Y	Z		
3	SFGdor.L	−22	18	72	−2.9722	0.004
4	SFGdor.R	24	20	70	−2.8655	0.006
5	ORBsup.L	−30	50	−14	−2.4309	0.008
29	INS.L	−40	0	−10	2.2595	0.040
42	AMYG.R	24	−2	−14	3.1447	0.010
45	CUN.L	−8	−76	20	−2.0810	0.009
46	CUN.R	10	−74	−20	−2.4647	0.048
89	ITG.L	−50	−40	−20	−2.3539	0.042
90	ITG.R	52	−38	−20	−2.7402	0.031

Note: AAL No., automated anatomical labelling number; MNI, Montreal Neurological Institute; SFGdor.L, left dorsolateral superior frontal gyrus; SFGdor.R, right dorsolateral superior frontal gyrus; ORBsup.L, left orbital superior frontal gyrus; INS.L, left insula; AMYG.R, right amygdala; CUN.L, left praecuneus; CUN.R, right praecuneus; ITG.L, left inferior temporal gyrus; ITG.R, right inferior temporal gyrus; The statistically significant level was set as *p* < 0.05.

## Data Availability

The data presented in this study are subject to privacy/ethical/legal restrictions and cannot be made publicly available. However, the data will be provided upon reasonable request to the corresponding author, in compliance with applicable regulations and ethical guidelines.
